# Reasons for Women's Choice of Elective Caesarian Section in Duayaw Nkwanta Hospital

**DOI:** 10.1155/2019/2320743

**Published:** 2019-07-07

**Authors:** Kennedy Diema Konlan, Elizabeth Kpodotsi Baku, Milipaak Japiong, Kennedy Dodam Konlan, Roberta Mensima Amoah

**Affiliations:** ^1^Department of Public Health Nursing, School of Nursing and Midwifery, University of Health and Allied Sciences, Ho Volta Region, Ghana; ^2^Department of Nursing, School of Nursing and Midwifery, University of Health and Allied Sciences, Ho Volta Region, Ghana; ^3^Department of Nursing, West End University College, Accra, Ghana; ^4^Department of Public Health, School of Allied Sciences, University for Development Studies, Tamale Northern Region, Ghana

## Abstract

**Background:**

Caesarean section is one of the most commonly performed major surgeries in obstetric practice intended to save the mother and child and in turn reduce maternal and perinatal mortality. The steadily increasing global rate of caesarean section has become one of the most debated topics in maternity care. This study identified the factors that influence women's choice of elective cesarean sections in the Duayaw Nkwanta Hospital.

**Methodology:**

This study used descriptive cross-sectional survey method to collect data from 78 post-caesarean section women above age 18 years. A whole population sampling method was used to trace respondents to their homes to administer a pretested questionnaire. Data was double-entered into Microsoft Excel spreadsheet, compared, cleaned, and transported to Statistical Package for Social Sciences (SPSS) version 21. Data was analyzed using descriptive statistics with a Pearson correlation test.

**Results:**

Post-caesarean section women (37.2%) indicated CS is a pain-free method of birth while 57.1% reported CS is safe for both mother and baby. Others (28.2%) chose CS based on a friend's advice and 19.2% on religious advice. The relationship between age of respondents and the number of times of having CS showed a weak positive correlation (r= .170, N= 78,* p*≤0.136, two-tailed test). There was a significant positive correlation between average monthly income of respondents and the number of times of having a CS birth (r= .320, N= 78,* p*≤ 0.004).

**Conclusion:**

It is imperative that there is heightened interest in educating mothers on associated benefit and risk of elective caesarean sections as a method of birthing by nurses and midwives in the antenatal clinics.

## 1. Introduction

Childbirth is a profound and powerful human experience. Women's accounts of birth often describe feelings of empowerment, elation, and achievement, particularly following vaginal birth without medical interventions [1]. Other women associate childbirth with trauma, loss of control, fear, pain, and anxiety [2]. The term “caesarean section” (CS) refers to the operation of delivering a baby through incisions made in the mother's abdominal wall and uterus. It is performed for certain medical indications such as placenta previa or transverse lie. Caesarean section can be a life-saving operation [2].

Caesarean section is medically indicated when a significant risk of adverse outcome for mother or baby is present if the operation is not performed at a given time [3]. The use of caesarean section for more vague medical indications and nonmedical reasons (for example, maternal request) is increasing in many resource-rich health services [3]. Non-medically indicated caesarean sections may be performed for reasons other than the risk of adverse outcome, if the person(s) assessing risk feel it is outweighed by the physical or psychological benefits. It has been suggested, for example, that a proportion of women, who request caesarean section for no apparent medical reason, may actually have been influenced by previous or current psychological trauma such as sexual abuse or a previous traumatic birth [4]. These may sometimes legitimately be regarded as clinical indications.

Cesarean section rate has increased in different parts of the world, both in developed countries and in developing countries [5]. The rates vary all over the world and researchers have examined whether nonmedical indications for caesarean section, such as obstetrician preference or maternal request, explain the regional variation [6]. A study by Danso et al. [6] found that preference for vaginal delivery among women who had delivered by caesarean section was high; however, the majority of these women had a generally positive opinion of caesarean section, as compared to vaginal delivery [6].

The phenomenon of patient-initiated elective caesarean section is a touchstone for these trends of increasing maternal choice of birth [4]. An estimated 18.5 million caesarean sections are carried out annually in the world, and in 3.6% the procedure is performed without any medical or surgical indications [7]. The World Health Organization (WHO) recommends caesarean delivery rates should not exceed 10-15%. WHO established that caesarean section is an essential treatment in pregnancy and is recommended at a rate of 5–15% of all births [8]. The caesarian section rate has increased in different parts of the world, both in developed countries and in developing countries [5]. Increases in cesarean sections worldwide have been well documented over the past two decades [4]. The increasing trend of CS has generated much controversy regarding the causes of such tendency [7].

A recent WHO publication reports that between 1990 and 2014 the global average CS rate increased from 12.4 to 18.6% with rates ranging, depending on region, between 6 and 27.2%, and rising at an average rate of 4.4% per year [9]. Epidemiologic studies have shown that, in high-income (HIC) and some low- and middle-income countries (LMIC) alike, CS is being provided at higher rates than recommended. The lowest rates were found in Africa (7.3%), followed by Asia (19.2%), Europe (25%), Oceania (31.1%), and North America (32.3%), with Latin America and the Caribbean having the highest rates at 40.5% [9]. While all the other regions showed an increase in CS, there was a small, but real increase in the CS rates in sub-Saharan Africa (SSA) over that time period [9].

In Ghana, only 4% of live births are by caesarean delivery [6]. A survey by Ghana Statistical Service (GSS) [10] reported that 11% of women who had a live birth in the two years prior to the survey delivered by CS [10]. The regional variations in CS are as follows: the highest was recorded in Greater Accra region, where nearly 1 out of 4 women (23%) had a caesarean section. The others are Volta (15%), Ashanti (12%), Eastern (12%), Brong-Ahafo (12%), and Central (11%) [6].

The percentage of births by caesarean section is an indicator of access to and utilisation of care during childbirth. It is estimated that between 5% and 10% of all births in a population will involve a complication that requires an intervention such as caesarean section [11]. Many pregnant women who have the option of choosing a birthing procedure opt for caesarian section. It has been suggested that the proportion of women who request caesarean section for no apparent medical reason may actually have been influenced by previous or current psychological trauma [2].

Various reasons in other jurisdiction have be ascribed for a caesarean section. There are maternal, foetal, and foetomaternal reasons for a CS. Ajeet, Jaydeep, Nandkishore, & Nisha, 2011, reported maternal causes (57%), foetal causes (30%), and fetomaternal causes (13%) [12]. Other factors that influence caesarean section are age of patients, the location of the hospital, prestige and financial capability, and late child delivery as well as emergencies. More patients prefer CS deliveries because they feel safe with the procedure [13].

**Figure 1:**
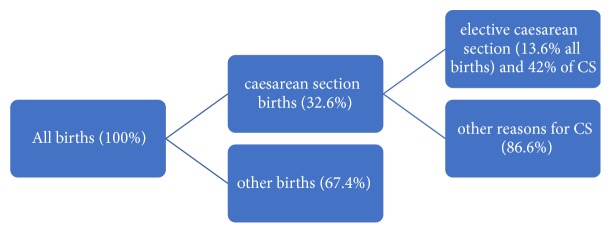
Flow chart showing deliveries and CS for the year 2017 at Duayaw Nkwanta District.

According to the Statistics Department of the St. John of God Hospital (Duayaw Nkwanta), in Tano North District, there were a total of 235 CS deliveries out of the 666 deliveries in the year 2016. This figure translated to 35.3% of the total annual deliveries. The rate of CS births in the hospital for the year 2017 is shown in Figure 1.

This study explored the reasons that influence women's choice of CS in the St. John of God Hospital in Duayaw Nkwanta.

## 2. Methodology

### 2.1. Study Design

This study was a descriptive cross-sectional study. The study participants were recruited in their homes once to respond to a questionnaire and no followup was made. The cross-sectional design was appropriate for this study because it involved the collection of data at one point in time (Polit & Beck, 2010)

### 2.2. Study Site

Tano North District is one of the twenty-two (22) districts in Brong-Ahafo Region. It was part of Sunyani District until 1989 when it was split to the Tano District. The Tano North District has its administrative capital at Duayaw-Nkwanta. It has a land size of about 700 Km^2^ and located on the Kumasi-Sunyani trunk road. The district shares boundaries with Sunyani Municipality to the North West, Asutifi District to the South West, Ahafo-Ano South District (in the Ashanti Region) to the South, Tano South District to the South West, and Offinso District (in Ashanti Region) to the North East.

The district is served by one hospital, six health centers, and two functional CHPS compounds with stationed midwives and Community Health Officers. About 85% of the road network in the district is not tarred. Some of these roads become unmotorable during the rainy seasons. This renders reaching out to various communities difficult. There are six subdistricts, namely, Adrobaa, Bomaa, Duayaw Nkwanta, Tanoso, Techire, and Yamfo, and a total of 126 communities with an estimated population of 100,792 in 2016.

### 2.3. Population

The study population included all women who underwent caesarian section in the Tano North District of the Brong-Ahafo Region. All CS deliveries in Tano North District are carried out in the St. John of God Hospital in Duayaw Nkwanta, the district capital. The target population included women who have had an elective CS birth in the year 2017 in the St. John of God Hospital. The women who have had CS births were 205 women in 2017. The proportion of women who underwent elective CS was 86 out of 205 representing 42%

### 2.4. Sampling

Total population sampling was used to select all the 86 clients who have undergone an elective CS in the St. John of God Hospital in Duayaw Nkwanta in 2017. Data of clients who have had elective CS were used to trace them to their various homes to administer the questionnaire after consent. In total 78 of the women had verifiable address and could be reached. All the women consented to take part in the study.

### 2.5. Data Collection and Analysis

Data was collected using a pretested research questionnaire. Data were collected from 30th August to 3rd September 2018. The questionnaires were designed into three parts, namely, demographics, socioeconomic characteristics, and factors influencing women's choice of CS birth.

The collected data was coded, double-entered into Microsoft Excel spreadsheet, cleaned, and transported into Statistical Package for Social Science (SPSS) version 21. The data was analyzed into descriptive statistics. Pearson correlation of women demographic characteristics and choice of CS birth was also made. A* p*-value equal to or less than 0.05 was considered as statistically significant.

### 2.6. Ethical Considerations

Ethical approval was given by University of Health and Allied Sciences Research and Ethical Committee of the Institute of Health Research (UHAS-REC A.10 [41] 17-18). Confidentiality of the responses was maintained strictly to ensure privacy.

## 3. Results

### 3.1. Demographic Characteristics

Table 1 showed majority of the respondents (44.9%) were aged from 28 to 35 years, 33.3% were 18 to 27 years, and 21.8% were in the age range of 36 years and above. On education, 83.3% had attended school or were currently in school as 41(52.6%) had tertiary education. In addition, 31(39.7%) had average monthly income below GH₵ 500, 20(25.6%) take average income of ₵1001 to 2000, 18(23.1%) take ₵ 501-1000, and 5(6.4%) had ₵2001 to 3000 as average monthly income. On source of healthcare financing, 70(89.7%) were beneficiaries of the National Health Insurance Scheme (NHIS). The respondents were asked whether they were informed or educated on any painless birthing method and 64(82.1%) reported to have no idea of any pain-free method while 14(17.9%) had knowledge of other methods classified to be pain-free. Among those who were informed of pain-free method of delivery, 10(71.4%) indicated CS, 5(35.7%) indicated epidural anaesthesia, and 3(21.4%) chose injection (opioid analgesia)

### 3.2. Factors Influencing the Choice for CS Birth

Table 2 shows information on factor that influences the choice of elective caesarean section. A proportion (37.2%) indicated CS is a pain-free method of birth. Some mothers (57.1%) reported they chose CS for being the safe method of delivery for both mother and baby. The perception of bad experience with previous spontaneous vaginal delivery (51.3%) chose CS delivery. Also 50% of the respondent find CS as more satisfying than spontaneous vaginal delivery. Other women (28.2%) chose elective CS based on a friend's advice that spontaneous vaginal delivery is painful. Following these experiences of CS, 30.8% had previous CS and were satisfied and hence wanted to repeat. For religious reason such as advice or prophecy from a priest 15(19.2%) chose to deliver by CS. Based on husband or partners advice 9(11.5%) chose to deliver. Due to emotional health such as stress and anxiety related to spontaneous vaginal delivery 41% chose elective CS without medical indication. Other reasons for CS included fear of complication for the baby (43.6%) and the fear of episiotomy (41%).

### 3.3. Sociodemographic Factors of Women and the Choice of Elective CS

From Table 3, it is shown that there was a significant negative weak relationship between number of times a woman had CS and the level of education (r= -.075, N= 78,* p*≤*0.*540, two-tailed test). Also, there was a significant moderate positive correlation between average monthly income of respondents and the number of times of having a CS birth (r= .320, N= 78,* p*≤0.004). This showed that higher levels of income are associated with high CS rate. The relationship between age of respondents and the number of times of having CS was examined using Pearson's coefficient. It was found that a weak positive correlation exists between the two variables (r= .170, N= 78,* p*≤0.136, two-tailed test). CS rate is increased as the ages of the respondent's increase. The relationship between parity and the number of times a woman had CS was investigated using Pearson's correlation coefficient. Results indicate a moderate positive correlation between the two variables (r= 0.391, N=78, p≤0.001, two-tailed test) with high parity associated with high levels of CS as in Table 3.

## 4. Discussion

The goal of this study was to determine the factors that influence the choice of CS delivery among post-elective CS women in the Duayaw Nkwanta District in the Brong-Ahafo Region of Ghana. The prevalence of elective CS rate in this study was calculated to be 35.3% of all births. The rate for elective CS was found to be 42% of all CS cases which is considered high as compared to the WHO range of 10%-15% of all cases.

### 4.1. Factors Influencing the Choice for CS Birth

Post-CS women (37.2%) described CS as a painless method of birth. With this knowledge the likelihood of having a CS birth is increased among those groups. Rosas [14] reported that one of the reasons women choose CS without medical indication is to avoid pain during delivery. In addition, Gosh and James [15] also reported that mothers who do not want to bear massive pain during labour have strong preference for CS. Zhao and Chen [5] reported that the fear of labour pain remains one of the most cited reasons for avoiding spontaneous vaginal delivery. This can be avoided when women are educated during antenatal clinic on birthing methods that are available and in which facility such services are accessed.

The condition or health status of the baby is another reason some women choose a CS birth. Some (57.7%) of the respondent had the contention that CS was safe for both mother and baby to avoid any complications. This indicates that some of these women have a heightened awareness or concern about the health of the baby. Locke et al. [11] reported in their study that the main reason for women delivery by CS was increased awareness for health of the newborn and the mother. This study is similar to other findings as in India, where CS is widely perceived as safer than spontaneous vaginal delivery for babies [16].

Post-elective CS women (51.3%) chose CS over spontaneous vaginal delivery because of traumatizing experience with the later. Women experiences directly influence their way of life or the choices they make. It is imperative that care providers ensure the women have a joyous experience in the laboring process. Oyewole et al. [13] reported that women who had traumatizing or bad experience with spontaneous vaginal delivery felt that pain of labour could only be voided through surgical procedures.

This study reported that 50% of post-CS women previously had spontaneous vaginal delivery. Comparing these two methods of birth, they all indicate CS was more satisfying. Oyewole et al. [13] indicated that women who have had both spontaneous vaginal delivery and CS are more likely to choose CS. The report further showed that some of the reasons are due to neighbourhood effect—woman in maternity department who sees how uncalmly the one with SVD in 2^nd^ stage cries and wails in pain [13]. In this study 28.2% chose to have CS without any medical indication based on a friend or family member's advice that spontaneous vaginal delivery is a painful experience. Preference for CS delivery is motivated by friends and family who suffered a lot due to spontaneous vaginal delivery [17].

Also, the study showed that 24(30.8%) who had previous CS prior to the latest one were satisfied and wanted to repeat the same procedure. According to a study by Oyewole et al. [13] women who had CS birth for whatever reason are likely to request CS in subsequent events. For religious reason like prophecy or advice by a priest (“*Okomfo”)*, malam, or prophet, 19.2% of post-elective CS chose CS mode of birth because they wished to have babies born in a particular ritual day or manner. Some of these women might have consulted the religious leader prior to the birth in other to determine the possible outcome of the pregnancy. A study in India by Manjulatha and Sravanth [18] reported that in areas where religious and religious beliefs take place prior to anything people listen to religious leaders and practice religious norms such as obeying prophecies and choosing to give birth on sacred day to receive blessing from God. In Ghana too, many times have we seen a couple choosing elective CS on a particular day just to name the child after a prominent figure of their family.

In this study it was reported that 11.5% of the post-elective CS women delivered by CS based on their husband or partners' advice. Phuong Thi Nho [17] reported in his study that CS is sometimes motivated by husband who had experience of watching their wives suffer during a spontaneous vaginal delivery. Also 16.7% of post-CS women chose CS birth with the aim of preserving their sexual organ and for resumption of sexual activity soon after birth. Some women have considered CS as a means to preserve their sexual function even though association between sexual problem and mode of delivery are yet to be substantiated [19]. However woman who had suffered perinea injury (Radestadd et al., 2008) and had episiotomy assisted vaginal delivery with history of dyspareunia [20] are more likely to delay resuming sexual intercourse after birth. However in another study, those who delivered by CS were on average likely to resume intercourse sooner than those who had vaginal delivery with episiotomy (Lurie et al., 2013).

Post-CS women (43.6%) also chose CS because of fear of complication for the baby. In many cases mothers want CS as this is safe procedure for the baby who is considered precious because of previous stillbirth or foetal analysis [21]. Some (41.0%) of the women fear episiotomy and opted for CS birth. Woman who had episiotomy assisted childbirth are late to resume sexual activity; they stand highest risk of getting infection and complication where there is failure to heal. They surfer dyspareunia and have reported decreased libido and widening of their vagina (Raddestadd et al., 2008).

### 4.2. Association between Sociodemographic Factors and CS Education

We found out that higher education tends to results in lower CS rates. In a study conducted by WHO (2014), most women who delivered by CS had a university degree. Another study reported that women and their spouse who chose CS delivery were educated in diploma and university level and there were no significant differences between groups who had education and medical field and those who do not (Abbaspoor & Noor, 2010). The contradiction in the findings may be due to sociocultural differences where the other studies were conducted.

It was also found that a significant moderate positive correlation exists between average monthly income and CS rate. This means when income increases, the likelihood of having a CS also increases. A study reported that CS is a conventional economic good, in the sense that the higher ones income, the more one is inclined to “purchase” [22]. In other studies, Brazilian women consider CS to be a higher class mode of birth, but Swedish women believed that requesting CS has a lower social class in terms of income than vaginal delivery [23]. We believe the ability to pay for the CS is a major contributing factor because the client wants it and can pay so why not to do it for her. As one's age increases, the person is more likely to choose CS birth than those with lower age range. This is true but has a weak correlation in this study. As maternal age increases, CS rate increases more than SVD. Another study points out that maternal age over 35 years is a factor contributing to the rise in CS without medical indication [22]. The relationship between parity and CS was moderate. Higher parity tends to results in higher rates of CS. In multiparous women, there are significantly higher CS rates than in nulliparous women.

## 5. Conclusion

Nurses and midwives should explain carefully the benefits and the possible risk/complication associated with CS to clients at antenatal clinic. All available birthing methods should also be explained giving the merit and demerits to the clients during antenatal clinic sessions.

The results of this present study could be applied in nursing and midwifery education by incorporating knowledge on the need to teach expectant mothers on birthing methods, their effects, and the need to choose wisely. This could also be done in a culturally competent and culturally congruent manner.

## Figures and Tables

**Table 1:** Demographic characteristics of respondents.

Demographic variable	Category	Frequency	Percentage (100%)
Age in years	18-27 years	26	33.3
	28-35 years	35	44.9
	36 years and above	17	21.8

Literacy	Yes	65	83.3
	No	13	16.7

Level of education	Primary	9	11.5
	Secondary	16	20.5
	Tertiary	41	52.6
	No formal education	12	15.4

Average monthly income	Below ₵500	31	39.7
	₵501-1000	18	23.1
	₵1001-2000	20	25.6
	₵2001-3000	5	6.4
	Above ₵3000	4	5.1

Religion	Atheist	1	1.3
	Christian	56	71.8
	Muslim	13	16.7
	Traditional religion	8	10.3

Ethnicity	Akan	39	50
	Ewe	14	17.9
	Northern	16	20.5
	Others	9	11.5

Marital status	Never married	12	15.4
	Cohabitation	7	9
	Married	52	66.7
	Divorced	5	6.4
	Widowed	2	2.6

Occupation	Student	7	9
	Unemployed	9	11.5
	Public employment	37	47.4
	Self-employment	17	21.8
	Housewife	3	3.8
	Others	5	6.4

Health insurance	NHIS	70	89.7
	Private health insurance	3	3.8
	None	5	6.4

**Table 2:** Factors Influencing the Choice for CS Birth.

Reason for CS delivery	Category	Frequency	(100%)
CS is painless method	Yes	29	37.2
	No	49	62.8

Safety of the baby	Yes	45	57.7
	No	33	42.3

Bad experience with previous vaginal delivery(ies)	Yes	40	51.3
	No	38	48.7

CS is more satisfying than spontaneous delivery	Yes	39	50
	No	39	50

Friends advised CS is painless	Yes	22	28.2
	No	56	71.8

Wanted to repeat CS	Yes	24	30.8
	No	54	69.2

Religious reasons	Yes	15	19.2
	No	63	80.8

Advice from spouse	Yes	9	11.5
	No	69	88.5

Perseveration of sexual function and early resumption of sexual activity after birth	Yes	13	16.7
	No	65	83.3

Emotional health	Yes	32	41
	No	46	59

Reduce complications by mother and baby	Yes	34	43.6
	No	44	56.4

Fear for episiotomy	Yes	32	41
	No	46	59

**Table 3:** Summaries of Pearson (r) correlation test.

VARIABLES	PEARSON CORRELATION (R)
Number to times of having CS	-0.075
Level of Education	

Average monthly Income	0.320
Number of times of having CS	

Age	-0.170
Number of times of having CS	

Parity	0.391
Number of times of having CS	

## Data Availability

The data used to support the findings of this study are available from the corresponding author upon request.
